# Zebra finch tutees not only share the melody but also the rhythm of their tutor’s song

**DOI:** 10.1038/s41598-025-22811-8

**Published:** 2025-10-13

**Authors:** Lara S. Burchardt, Judith M. Varkevisser, Michelle J. Spierings

**Affiliations:** 1 Humboldt-Universität zu Berlin, Berlin, Germany; 2 Leibniz Zentrum für Allgemeine Sprachwissenschaften Berlin, Berlin, Germany; 3 Radboud University Nijmegen, Nijmegen, Netherlands; 4 Institute of Biology Leiden, Leiden University, Leiden, The Netherlands; 5 Leiden Institute for Brain and Cognition, Leiden University, Leiden, The Netherlands; 6 Department of Behavioral and Cognitive Biology, Vienna University, Vienna, Austria

**Keywords:** Rhythm, Ontogeny, Isochrony, Song acquisition, Developmental biology, Evolution

## Abstract

Recent studies have highlighted rhythmic patterns in the vocalizations of several animal species, particularly in songbirds. Whilst it is known that for instance zebra finches copy the spectral content of their tutor’s song, little is known about possible rhythmic copying. In this study, analyzing the inter-onset intervals of the elements in the motifs of 17 tutors and their 37 tutees shows that rhythms are most similar between tutors and their respective tutees – though only when considering the whole motif produced by the tutee. When examining only shared or only non-shared elements, rhythmic patterns differed. This suggests that the shared rhythm goes beyond copying the temporal features of individual elements; tutees perhaps adjust the rhythm of non-shared intervals, resulting in an overall rhythm resembling their tutors’. Furthermore, tutees who share all elements tend to sing slower, more consistent rhythms, while those who improvise more in elements or melody show greater variability in both rhythmic rate and consistency. This might hint at two distinct evolutionary strategies: one favoring consistent, slower rhythms, the other favoring novelty. Female choice experiments might be the next step to prove this hypothesis.

**Supplementary Information:**

The online version contains supplementary material available at 10.1038/s41598-025-22811-8.

## Introduction

Rhythm is a fundamental component of musical structure. It is the systematic patterning of sound items in a string and is characterized by a nonrandom, ordered, predictable, and recurrent alternation of sound and silence in a temporal sequence^1,2^. Although musicality might be a uniquely human feature, the production of rhythmic movements or vocalizations is shared amongst many species. For example, several mammals show a rhythmic pattern in their movements and vocalizations^3–5^, and different forms of complex rhythms are known to occur in birdsongs^6–8^.

Rhythmic patterns can be periodic, with regular intervals, or aperiodic with various interval durations between the sounds that still adhere to an overall repeated structure. Isochronous patterns, where each interval is of equal duration, represent a specific type of periodic rhythm. In many musical traditions, including Western European and African music^2,9^, timing is often anchored around an isochronous beat, a cognitive construct that listeners perceive even when it is not directly present in the acoustic signal^9,10^. This beat is often organized within a metrical structure, a hierarchical patterning of strong and weak beats that enables listeners to discern the beat and the underlying rhythmic structure. Isochrony is the most common rhythm across all cultures, as well as the most profound rhythm found in the vocalization of other species^11,12^. This might be caused by certain physical or cognitive constraints that lead to the production of these highly regular rhythms, or it might enhance vocal interactions or vocal learning^6,13^.

Some of the strongest examples of isochrony in animal movements and vocalizations come from songbirds^14–18^. Various bird species exhibit coordinated vocal behaviors such as chorusing^19^ and synchronize movements with song during courtship displays^20–23^. Zebra finches (*Taeniopygia guttata*) display synchronized song and dance during courtship, although it remains unknown whether they coordinate singing across individuals^24,25^. Similar to human music and dance, an isochronous beat may also support coordination in birds. Benichov and colleagues (2016) demonstrated that zebra finches could align their unlearned calls with a robot producing isochronous timed calls, suggesting an ability to predict regular call patterns^26^. This synchronization appears reliant on the forebrain motor pathway used for song production, highlighting complex timing skills in both male and female zebra finches despite differences in their song systems.

Rhythmic templates serve as a foundation for song learning, particularly in vocal learning species, such as zebra finches. Isochronous sequences, characterized by predictable temporal grids, facilitate vocal experimentation and learning by anchoring vocal emissions to rhythmic onsets^27^. In songbirds, this mechanism enables the acquisition of melodic templates, which in turn reinforce rhythmic segmentation and regularization, creating a dynamic interplay between rhythm and melody^28,29^. Such processes may be especially relevant during social interactions, such as chorusing and turn-taking, where rhythm and melody bootstrapped each other over evolutionary time^30–32^. The hypothesis that rhythmic isochrony supports vocal learning and the acquisition of rhythm and melody^33^ provides a valuable framework for addressing knowledge gaps in vocal rhythm and its communicative and cognitive roles. Zebra finches, with their well-studied vocal learning capabilities, offer an ideal model for investigating these processes, which parallel human rhythmic and melodic integration.

Whilst in zebra finch vocal learning, most studies have focused on the spectral features of the song^34,35^, the temporal structure is just as relevant. For example, zebra finches display clear isochronous rhythms in their songs^7^, which might even extend to regular intervals between song bouts^36^. Moreover, the temporal aspects of songbird vocalizations show a clear developmental aspect. In zebra finch song, the temporal organization of song syllables and the silent gaps between syllables increasingly become more rhythmic throughout the first year of life^37^. Also, towards adulthood, these rhythmic patterns become more stereotyped than they are in the earlier stages of song development^38^. Experiments show that rhythms in songs may function as a grid to maintain the spectral structure of the elements^28^. Zebra finches learn their vocalizations from a male tutor, usually their father. The song learning process can be roughly divided into two stages: the sensory acquisition phase at ~ 20–65 days post-hatching, where the bird forms an auditory template without producing full songs yet, and the second, sensory-motor phase at ~ 30–90 days post-hatching, where the bird starts producing a song based on that template. After 90 days they produce a crystallized song, consisting of several repeated motifs, that stays unaltered throughout their lives^39^. During this learning period, the isochronous rhythms of the tutor song might play an important role in the development of the tutee’s song. Java sparrows are known to learn the temporal features of their songs, such as song duration and tempo, from their fathers^40^. Also in zebra finches, the durations of silent intervals between song motifs are similar between tutors and tutees^41^, suggesting a more important role of temporal precision in song learning than previously assumed. However, it remains unclear whether and how the rhythm within a motif, which is isochronous but individually distinct in zebra finches, is copied^7^.

In this study, we focued on the consistency with which the rhythm of zebra finches is copied from their tutor by young adult males. We recorded 17 tutors and 37 tutees, after song crystallization, and analyzed the beat patterns in the element structure of their vocalizations. The comparisons in rhythm between the tutor and the tutees were based both on the whole motif (everything) as well as on the parts of the motif that were shared between tutor and tutee (all shared/part-shared), as zebra finches do not always copy all elements from the tutor. Finally, the analysis also separately compared the parts of the song that were new in the tutees’ sequences and did not occur in the tutor’s song (not-shared). By measuring four rhythmic parameters (the inter-onset-intervals (IOI’s), the normalized pairwise variability index (nPVI), the coefficient of variation, and IOI beat), we gain a better understanding of the processes during vocal learning regarding the rhythmic structures of the motif.

**Fig. 1:**
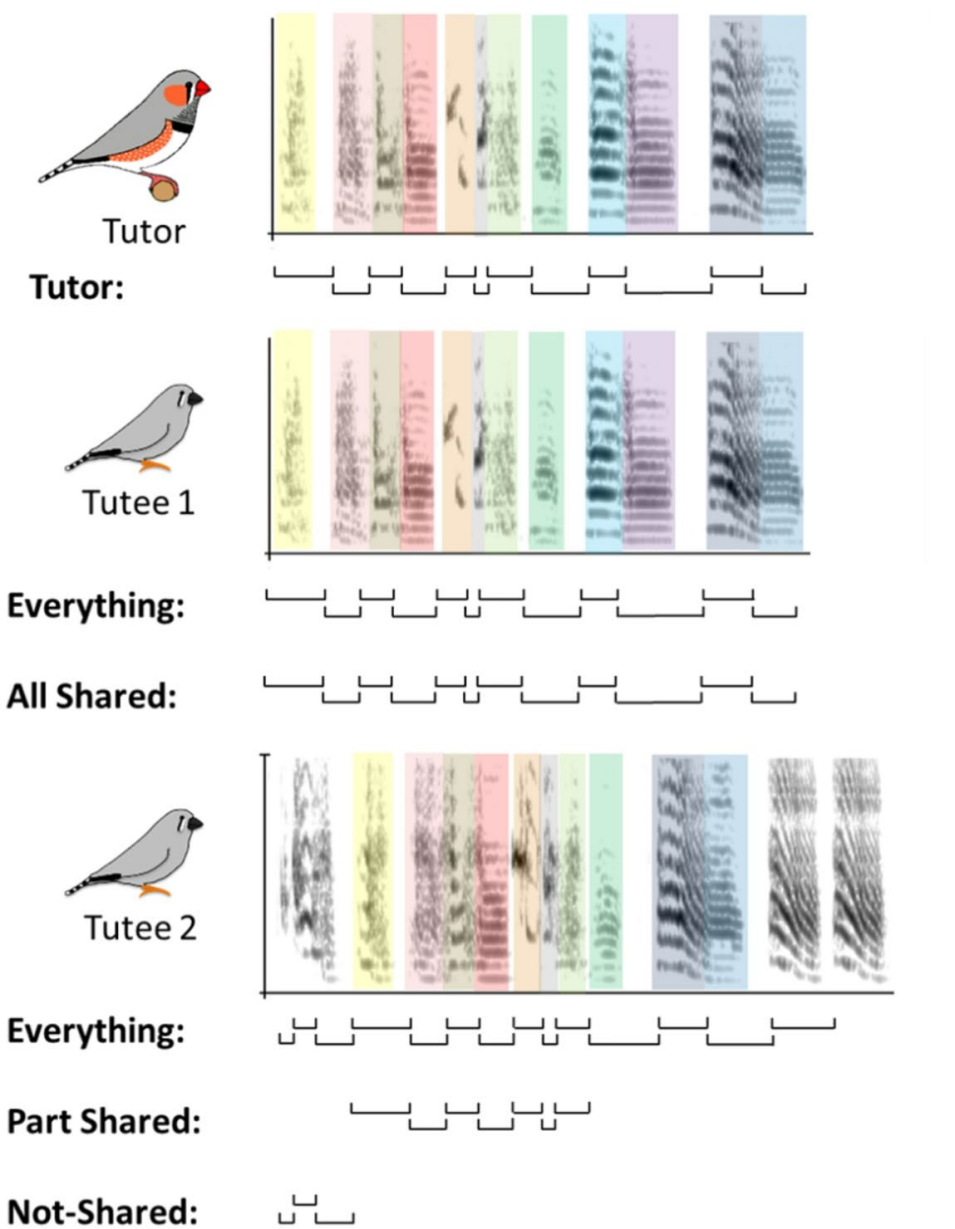
Examples of the spectrogram for a motif of a tutor and two of its tutees; one tutee (1) copied the tutor’s sequence completely and one tutee (2) copied part and improvised part of its sequence. Colored tutee elements were considered to be copies of the same colored- elements in the tutor’s motif. The lines below the spectrograms indicate the inter-onset-intervals that were included in the different subsets (Tutor, Everything, All Shared, Part Shared, and Not Shared) in our analysis. Note that only sequences of at least three consecutive intervals were included in the subsets (see Methods).

## Materials and methods

### Subjects

Subjects for this study were 54 male zebra finches (17 fathers (tutors) and their sons (tutees)) from the Leiden University breeding colony. The number of chicks per breeding pair ranged from 1 to 4, with a median of 2 (S Table 1). Breeding pairs were housed in breeding cages (80 × 40 × 40 cm), with other pairs audible, but not visible to them. Chicks remained in the breeding cage with their parents, until approximately 65 days post-hatching (dph), when they were moved to single-sex aviaries.

Throughout, all birds had *ad libitum* access to food and water. The chicks whose song was analyzed in this study were not raised specifically for this study, and their parents had not been selected according to any specific criterion other than having a within-pair coefficient of relation smaller than 0.125.

### Recordings

Between 99 and 192 dph, i.e. after song crystallization, the tutees were moved individually to a recording cage (70 × 30 × 45 cm) in a sound-attenuated chamber (125 × 300 × 250 cm). A Sennheiser MKH40 microphone (Wedemark, Germany) suspended 0.5 m above the top of the cage and ISHMAEL software (v. 1.0.2, http://www.pmel.noaa.gov/vents/acoustics/whales/ishmael/; automatic energy detection settings for 2000–10000 Hz, detection threshold 1, detection limits 0.2–100 s, buffer 3 s) were used to record the birds’ songs. The birds remained in the sound-attenuated chamber for up to 24 h. If they had not produced any song during this period, they were moved back to their home cage, and a new attempt at recording them was made the week after. All birds were housed individually during the recording sessions, ensuring all recorded songs were undirected.

### Song selection and annotation

For all sound editing and annotation, spectrograms were calculated with the Praat software (fast Fourier transformations with 1000 time and 250 frequency steps, 0.005s window length, dynamic range 55 dB, Gaussian window, Praat v. 6.0.19, Boersma & Weenink, 2008). For each bird, ten songs were randomly selected from the 24-hour song recordings. A song was defined as a series of motifs separated from other vocalizations by more than two seconds of silence or a series of motifs preceded by multiple introductory notes^42^. From each song, the first motif was cut out of the sound file using Praat. Introductory notes that did not occur with every repetition of the motif were not considered part of the motif and therefore not included in the analysis.

Using TextGrids, the onset of each syllable and element in the motif was then annotated. Syllables were defined as sounds separated from other sounds by at least 5 milliseconds of silence. Elements were defined as sounds separated from other sounds by either silence or by an abrupt change in frequency or structure^43^. One syllable could thus consist of one or several elements. For the tutor’s motif, each unique syllable and element was labeled alphabetically. For the syllables and elements in the tutees’ motifs, we assigned the same letters as the syllables and elements in the tutor’s motif that they resembled in frequency pattern, duration, overall shape, and sequential position. We assigned different letters to tutees’ syllables and elements that did not resemble any of the syllables and elements in the tutors’ motif.

### Analysis

Rhythm analysis was carried out on the level of elements using the workflows described in Hersh et al. and Burchardt & Knörnschild^44,45^. We had 17 tutors and 10 motifs for each tutor, the tutor motifs were always analyzed as a whole. For 37 tutees we also had 10 motifs each, which were analysed in three different ways (Fig. 1). First, the whole motif was analyzed (indicated as “everything” in figures); second, only the sequences of elements in the motif shared with the tutor were analyzed on the condition that it was 3 or more elements long (all-shared and part-shared). All-shared sequences indicate that the tutee copied all elements from the tutor in the same order, potentially only adding more elements at the end. Part-shared indicates a snippet from the beginning of a motif, that follows the tutor’s elements sequence but incompletely. We then only analyzed the part, that was shared with the tutor. Third, we analyzed the not-shared elements of the motif on the condition that there were 3 or more elements in a row. While for cases 1 and 2 we would have a maximum of 1 sequence per motif for the analysis, in the third case we could have several sequences per motif for analysis if in the whole element sequence of a motif several sub-sequences of at least 3 adjacent not shared elements existed. Rhythm analysis was based on inter-onset intervals, the duration between the start of one element and the next element. We calculated the following rhythm parameters for every motif/analysis sequence: (1) coefficient of variation (adjusted for small sample sizes), (2) normalized pairwise variability index, (3) IOI beat in Hz (as in beats per second) using the mean IOI duration of the sequence as basis. The coefficient of variation is the standard deviation divided by the mean, to make it comparable, independent of the mean. The normalized pairwise variability index is a local measure, indicating how well the next IOI can be predicted based on the previous IOI. The nPVI would be 0 in a perfectly isochronous sequence. Higher nPVI values correspond to a higher variability between IOIs. The IOI beat is calculated by dividing 1 by the mean IOI duration of a sequence, transforming it into a frequency in Hz, as in beats per second. Calculations are detailed in Burchardt et al. and Hersh et al.^44,45^.

### Statistics

When comparing the different groups of data (tutor, tutee everything, tutee part-shared, tutee all-shared, tutee not-shared) we calculated Welch’s T-test adjusted for multiple testing (Bonferroni correction) and the effect size Cohen’s D. Furthermore, we calculated Pearsons correlation coefficient r for the relationship between the rhythmic parameters and number of elements in the sequence.

Given the exploratory nature of this study, our focus was on descriptive and comparative statistics to identify general trends in rhythmic structure across individuals and groups. While the data include repeated measures (multiple sequences per bird) and a nested structure (tutees within tutors), we intentionally avoided fitting mixed-effects models. This is because our analysis subsets overlap (e.g. ‘everything’, ‘all-shared’, ‘not-shared’), and including them all simultaneously in hierarchical models would violate the independence assumption. Additionally, the uneven distribution of tutees per tutor and the post hoc nature of group categorisation limit the suitability of inferential modelling. Instead, we employed Welch’s t-tests (adjusted for unequal variances and multiple comparisons) and calculated effect sizes (Cohen’s d) as descriptive tools. These were complemented by full visualisations of the data distributions to aid interpretation. All statistical tests were treated conservatively, and we aimed to describe and compare observed rhythmic patterns, not to explain mechanistic causality.

### Software and code

The rhythm analysis was run with the RANTO app from GitHub (https://github.com/LSBurchardt/R_app_rhythm/tree/master/RhythmAnalysis*).* All codes for pre- and postprocessing as well as analysis can also be found on GitHub: https://github.com/LSBurchardt/ZebraFinch_LearningRhythm.

## Results

**Fig. 2:**
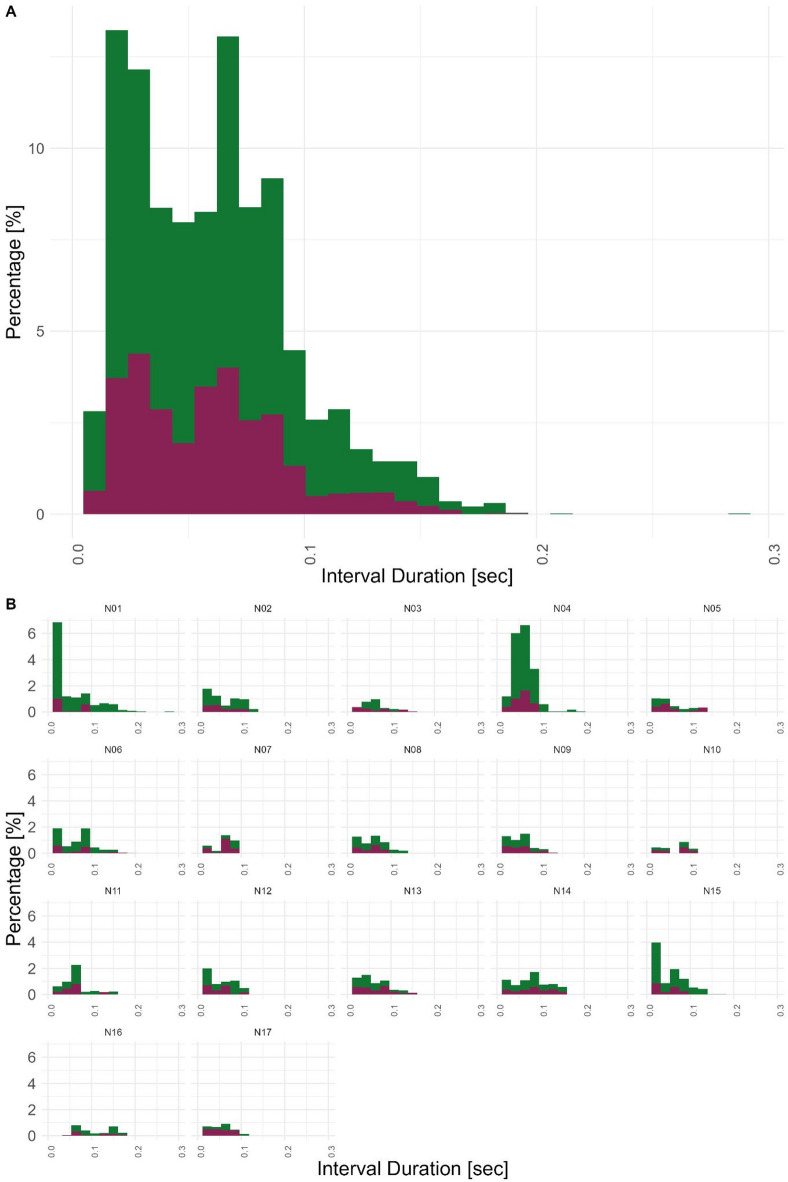
IOI distributions across the dataset. (**A**) Distribution of Interval Durations of the whole dataset, tutors in dark red (in the forefront) and tutees in dark green (in the background). Both distributions have the same slightly bimodal shape. (**B**) Distribution of IOIs from tutor and tutees divided by nests. Tutee distributions follow the tutor’s IOI distribution strongly. The Percentages of all subplots and both tutors and tutees combined sum up to 100%.

We analyzed the rhythmic structure in the song of a total of 54 male zebra finches, 17 adult male tutors (17 nests), and their respective 37 tutee sons. To find out if they copy not only spectral aspects of their tutors’ songs but also the temporal aspects, we calculated rhythm parameters on ten motifs of each individual. As a first indication of temporal copying, we analyzed the distribution of inter-onset intervals, the duration from the start of one element to the next element. A total of 4839 intervals between song elements overall ranged from 0.009 s to 0.29 s with a mean of 0.06 s and form a slightly bimodal distribution in both tutors and tutees, the first peak likely shows intervals between elements within one syllable, the second peak, on the other hand, likely shows intervals between syllables (Fig. 2A). Distributions can look quite different between nests, though. The shapes of the distribution of tutee elements follow the shape of the distribution of tutor elements very closely (Fig. 2B). This visualization served as a descriptive starting point and initial motivation for our more detailed quantitative comparisons presented in the following sections.

**Fig. 3:**
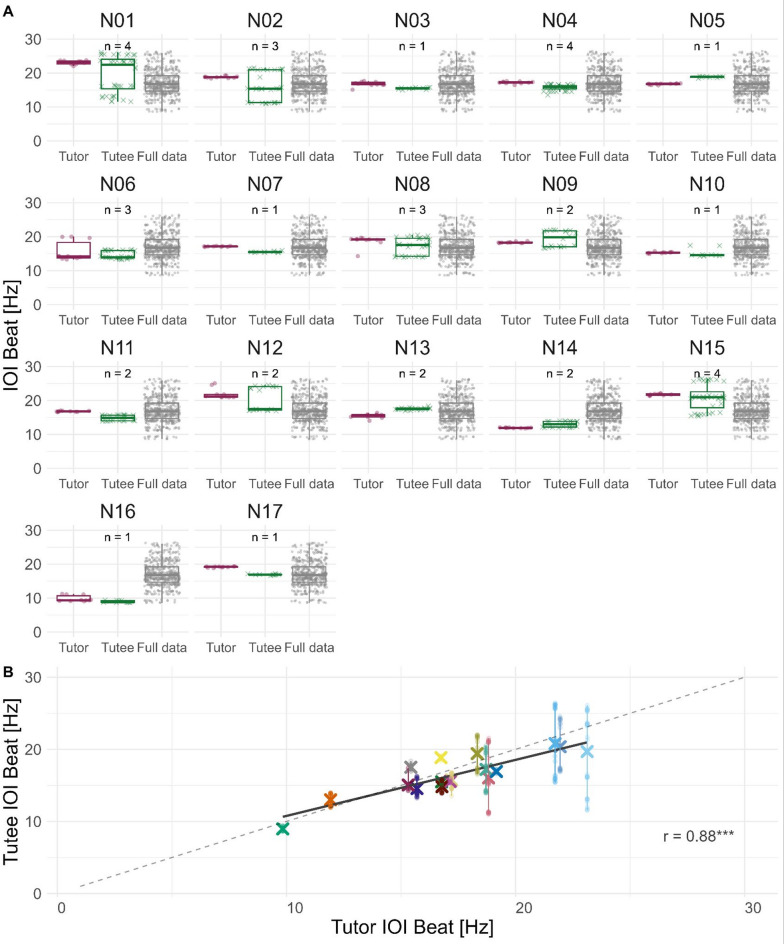
IOI Beat copying per Nest. (**A**) Tutor of the nest, Tutees of the nest, and as a third comparison the IOI beat of the full dataset (all tutors, all tutees, full sequences only). The number of tutees per nest is shown across the tutee group. We can see that often the tutor and tutee of a nest are closer to each other than to the full data. This is especially clear in Nest N01, N15, and N16. In nests, where the tutor is similar to the full dataset median, this is not as visible. (**B**) The mean of each tutor’s IOI beat is plotted against their tutees’ mean IOI beat per nest (large X, different colors indicate different nests), and all tutee rhythms are individually shown as points. Tutee rhythms follow their tutor’s rhythms but tend to be slightly slower. The dotted grey line indicates a theoretical perfect match of tutor and tutee rhythms, the dark, solid grey line shows the correlation of the data with a correlation coefficient of *r* = 0.88 (Pearson’s correlation, *p* < 0.0001).

Based on these interval distributions and the literature^7^ it could be assumed that we can model element onsets with an isochronous, that is metronome-like, rhythm. Using the IOI approach, we calculated best-fitting rhythm frequencies based on the mean of intervals in a sequence^45^. We find rhythms between 8.6 Hz and 26.4 Hz, with a mean of 17.1 Hz (sd: 3.7 Hz). The coefficients of variation of interval durations within a sequence range from 0.1 to 1.1 with a mean of 0.57 (sd: 0.15). We try to answer the question if rhythm parameters are more similar between the tutor and tutee(s) of a single nest than between all analyzed birds to see if an effect of the tutor on the rhythm production of its tutees might be observable. This is nicely illustrated in Fig. 3. There we see the found IOI Beats for every nest divided into tutor and tutees as well as all IOI beats calculated in the overall dataset (Fig. 3A, from left to right, dark green, dark red, and grey). As the found IOI beats overall form a normal distribution, we would assume that many individuals independent of their nest ID share similar IOI beats. This is true, but the nests deviating from this are especially interesting: for example, we have Nest N06, N14, and N16, where both tutor and tutee produce a much slower rhythm than the group median. In nests N01 and N15, we can see the opposite, that both tutor and tutees produce much faster rhythms than the group median. Furthermore, if we compare the mean Tutor IOI beat with the respective tutee IOI Beats, we see that tutees’ rhythms cluster around their tutors’ rhythm, though tutees seem to be slightly slower on average (Fig. 3B). This is also evident in a strong correlation of *r* = 0.88 (Pearson’s correlation coefficient, *p* < 0.0001).

**Fig. 4:**
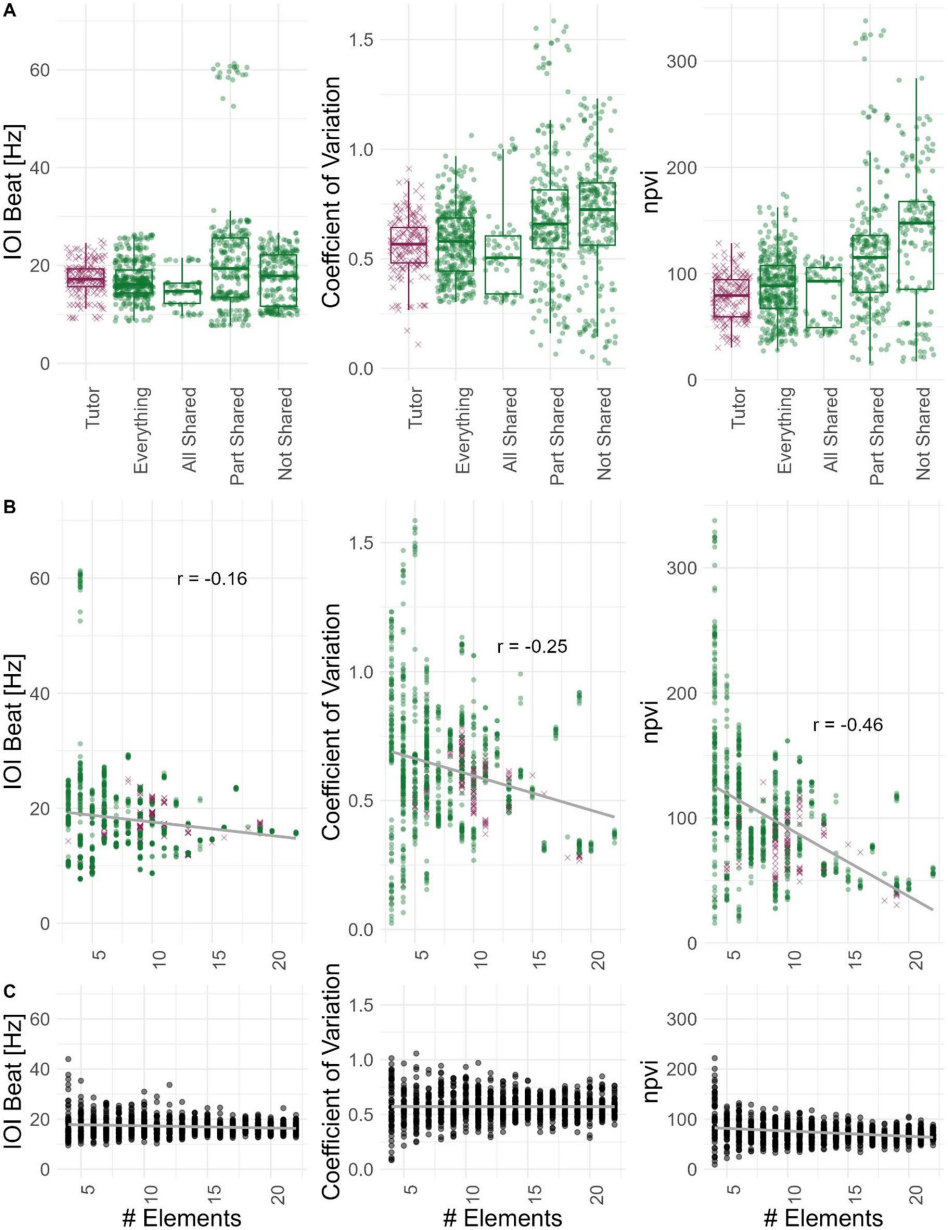
Rhythmic parameters across nests. (**A**) Per Group: IOI Beat per group. Coefficient of Variation per group. nPVI per group. (**B**) Correlation with the number of elements in the analysis unit, tutors, and all groups of tutees are considered here: everything, all-shared, part-shared, and not-shared. (**C**) Theoretical correlation between parameters and number of elements (1000 random sequences drawn from iois from dataset, min of 4 elements, max of 22 elements).

We proceeded to see if rhythms differ between tutees that share more or less elements with their tutor. Copying of the elements and their sequence from the tutor is not perfect and while there are tutees that copied the entire element sequence of their tutor correctly, there are many tutees who only copied the sequence in parts, adding or skipping elements in the process (Fig. 1). Therefore, we split the data and re-ran the rhythm analysis on the following subsets of the tutee songs: only considering the shared elements, which could either result in the tutee sharing all or part of the tutor song, and only considering not shared elements. The resulting sequences needed to be at least 3 elements long. Only consecutive elements were taken into account, which led to a situation where for the not shared elements, we could end up with more than one sequence per tutee motif. The resulting rhythm parameters for the different groups are shown in Fig. 4A. Rhythm parameters differ between the groups to different degrees.

We again ran correlation analyses on the different groups between the mean tutor IOI Beat [Hz] and mean tutee IOI Beat [Hz] in the different groups. We find the strongest correlation between tutor mean and tutee mean for sequences that share all elements (*r* = 0.94, *p* = 0.01). Correlations between tutor mean and the “part shared” and “not shared” sequences are much weaker (*r* = 0.63, *p* = 0.01 and *r* = 0.58, *p* = 0.05).

T-tests and effect size calculations between the groups showed relevant differences for example between the nPVI values of tutors and the whole sequence of tutees (everything, *p* = 0.0003, D = -0.35), while the IOI beat and the coefficient of variation do not differ significantly between these two groups (IOI Beat: *p* = 1.00, D = 0.09; CV: *p* = 1.00, D = -0.07, reported p values are adjusted for multiple testing). This is especially interesting as IOI Beat and CV are taking the whole sequence into account, while the nPVI is based on pairwise comparisons of intervals in the sequence, so locally higher variability as indicated in a higher nPVI does not necessarily impact the overall variability and rhythm or even more so, might actively work towards keeping the rhythm similar independent of local changes. Tutees overall show similar rhythmic parameters, but especially as they change certain elements compared to the tutor’s sequence, the individual intervals get more variable, also stressed by the fact, that nPVI does not differ significantly between the tutors and the tutees’ song, which share all elements (*p* = 1.00, D = -0.05). This local variability gets more prominent with changes in the element sequence. The three strongest effects (based on Cohen’s D) were all found for the nPVI between Tutor and not-shared element sequences, whole tutee sequences (everything) and not-shared elements, and between tutee sequences that shared all elements (all-shared) and not-shared sequences. For all three comparisons, the nPVI is higher, indicating more variability between intervals for the not-shared elements. The temporal structure in those individually improvised parts is more variable on a local level. Moreover, four comparisons are significantly different for all three parameters: the IOI beat, CV, and nPVI. This is the case for tutor vs. part-shared, everything vs. part-shared, all-shared vs. part-shared and all-shared vs. not-shared. Again, this indicates that rhythmic parameters differ more, the more the tutee’s sequence deviates from the tutor’s sequence. Detailed statistics for all comparisons can be found in the Supplementary Materials (S Tables 2, 3 and 4).

Lastly, we checked how the differences we find between the rhythmic parameters in the different groups relate to the number of elements in a sequence, as tutor sequences, whole sequences, and all-shared sequences are consistently much longer than part-shared and not-shared sequences. We find that indeed, all parameters decrease with an increasing number of elements in a sequence to different degrees (Fig. 4B, S Table 5). This is a property of the data at hand and not an inherent correlation between the parameters and sequence length. To establish that, we modeled random sequences between 3 and 20 elements (mimicking the found sequence lengths) purely made up of intervals we found in the analyzed sequences. Theoretically, we find no correlation between the rhythm parameters and the number of elements in a sequence (Fig. 4C).

Consequently, it seems to be an inherent property of the part-shared and non-shared sequence parts that they are slightly faster and mostly much more variable, i.e., their rhythm is more inconsistent.

Considering that we also analyzed the whole sequence of tutee songs, which incorporate all the smaller part-shared and not-shared snippets, it seems that overall tutees compensate for higher variation in some of the snippets, to end up with a rhythm similar to their tutors’ rhythm. Overall, the sequences of tutees that shared all elements are the slowest with on average the lowest coefficient of variation, i.e. showing the most stable quasi-isochronous rhythms.

## Discussion

In this study, we investigated to what extent not only the spectral parts but also the rhythm of zebra finch songs are learned or copied from a tutor. We find evidence that indeed an underlying metronome-like rhythm in tutee song is copied from tutors. The similarities are already apparent when only visually comparing distributions of interval durations between elements of motifs (Fig. 2B). Rhythm analyses calculating metronome-like rhythms for every motif further support this. Overall rhythms are more similar between a tutee and its tutor than compared to the whole dataset (Fig. 3). This is only true when considering the whole motif sung by the tutee, made up of shared and not shared elements. Especially analyzing subsets of the motifs whose sequences are innovated to differing degrees show significantly different rhythms, compared to the tutors’ rhythms. When considering the whole motif of a tutee, whether all shared or innovated to differing degrees, these differences diminish, though, suggesting that the shared rhythm is not a mere byproduct of element copying on the spectral domain, but a desirable outcome.

Interestingly, we see two different trends in the copying connected to the copying of elements in the spectral domain. Tutees can copy the exact sequence of elements from the tutor motif, but more often they slightly change the order or “improvise” fairly different element sequences. Linking that back to the rhythmic properties of the songs, two different strategies seem to emerge. Tutees who copy more elements exactly in the tutor sequence show more consistent and significantly slower rhythms, while tutees who “improvise” more on the spectral domain show much higher variability in the temporal domain. Considering female choice and that male song is a fitness-relevant trait^46^ we speculate that both a consistent, slow rhythm and an innovative element sequence are aspects favored by females. Those features might be mutually exclusive, meaning that it might not be possible for a bird to have both a very slow consistent rhythm, while also singing a very innovative element sequence. This could be leading to the two outcomes we see. Female choice experiments could shed further light on this hypothesis in the future.

The rhythms we found ranged from ~ 10 to 25 Hz, which is comparable, though on the lower end of rhythms analyzed in a previous study that found metronome-like rhythms mostly between 10 and 40 Hz^7^. In that study, rhythms were based on onset intervals of syllables (there called notes) but it was reported that the found rhythms also fit “gesture” onsets, which is what we call elements here. The rhythms were calculated with a slightly different method and the variability of intervals can not be compared between these two studies.

We can compare the variability parameters with studies from other exemplary animal vocalizations though. In the very rhythmic echolocation call sequences of two bat species and a sperm whale, mean coefficients of variation were found to be 0.23, 0.19, and 0.14, respectively, which is much lower than what we found here in zebra finches, though in a monosyllabic system^45^. The calculated values in zebra finches (mean CV 0.57) are comparable to values found in fish. In the /Kwa/ sounds of *Scorpaena spp*., a median CV of 0.8 was found in a rhythmic sequence type, though with a slightly more complex structure than signal isochrony. While in this example all element onsets fit well with an underlying metronome-like rhythm, not every beat in this theoretical rhythm is matched by an element^1^. This might in part be true for the zebra finch rhythms as well. While the variability we find in the data at hand is higher than in some other communication signals described as clearly isochronous, it still fits animal communication signals that have been described as rhythmic and are well described by an underlying isochronous rhythm. And while the variability is an interesting aspect for further consideration, to better understand rhythm development and its role in the context of acoustic rhythms serving as a fitness signal, the variability we find here, does not affect the general result we find. While the variability should be further studied, the result that overall rhythms of tutees very well follow their tutors’ song remains untouched.

This study was designed to explore rhythm copying in the songs of zebra finches within a descriptive and comparative framework. We aimed to characterise the broad patterns of how learners’ rhythmic structures align with those of their tutors, particularly when comparing full motifs with shared and novel sub-sequences. Due to the complex and overlapping structure of our dataset and the fact that it was not designed to test specific rhythmic hypotheses using inferential models, we decided against applying linear mixed-effects models. In particular, the different subsets of songs analysed for each bird (‘everything’, ‘shared’, ‘not-shared’) are not mutually exclusive and therefore cannot be modelled as independent observations. Instead, we relied on visual inspection and group-level comparisons using descriptive statistics and effect sizes to identify meaningful differences and trends. While this approach limits the scope for inference, it enables us to identify significant rhythmic patterns that can inform future, more specific investigations employing experimental designs that are more suitable for formal modelling.

Our findings underscore the importance of rhythm transmission during vocal learning in zebra finches, and, more specifically, show that this is relevant to the rhythmic features of zebra finch songs. As shown previously, the temporal structure of zebra finch songs becomes increasingly stereotyped during the sensory-motor phase of development^37,38^. The similarity in rhythmic parameters between tutors and their tutees, particularly in the part-shared song elements, highlights the precision with which rhythmic features are transmitted during song learning.

The observed overlap in rhythmic parameters may have a genetic component since the tutees in this study were the biological sons of the tutors. In Bengalese finches, genetic background influenced the accuracy in copying of temporal features, with more accurate learning of the tempo of the song when the song was produced by an individual with more genetic similarities to the tutee^47^. However, studies with cross-fostered zebra finches showed no influence of genetics on temporal song learning^41^.

The gradual development of rhythmic precision in zebra finches shares striking similarities with the acquisition of rhythmic and temporal patterns in human speech and music^2,29^. Both species exhibit a learning trajectory where temporal structures become increasingly stereotyped and refined, underscoring the shared cognitive and neural underpinnings of vocal learning across distantly related taxa^48^. However, unlike humans, zebra finches likely lack beat perception or the ability to organize rhythms hierarchically. This distinction aligns with broader findings in the field of comparative cognition, which suggest that beat perception is a rare trait among nonhuman animals, primarily observed in species capable of vocal mimicry^10^. The absence of beat perception in zebra finches may limit their ability to discern or replicate complex metrical structures, further highlighting the differences between their rhythmic capabilities and human musicality.

While this study focused on isochronous rhythms, our findings reveal the need for further exploration of variability and individual differences in zebra finch song rhythms. Investigating other rhythmic parameters, employing alternative analytical approaches, and extending this work to other populations or species will be crucial to further our understanding of rhythmicality in vocal learning. This will also give further insight into the hypothesis set by Ravignani (2021) that isochronous patterns can act as a grid for vocal learning of melodies^33^. The two main strategies of the zebra finches in the current study, either copying their tutors’ song elements more precisely but slowing down the rhythm, or copying fewer elements but having a more similar tempo, show that there is a link between spectral and temporal copying. Rhythms are not copied equally accurately by all zebra finches, rather, it depends on the accuracy of element copying. This supports the hypothesis that rhythm might aid spectral precision.

To conclude, in this study, we have shown that the rhythm in the song of zebra finch tutees is more similar to that in the song of their tutors than in the song of other conspecifics, but only when considering both shared and non-shared intervals. This suggests that rhythm might be copied, and that shared rhythms might not merely be a result of imitating the temporal properties (i.e., duration) of copied elements. These findings imply that zebra finches not only copy the spectral but also the rhythmic features of their tutors’ song. We suggest that female preference affects both rhythm and melody copying in zebra finches. We hypothesize that females prefer songs with a consistent and slow rhythm and with an innovative element sequence. Judging from our results, these two might be.

mutually exclusive, leading to a dichotomy of rhythmic consistency vs. melodic novelty. Moreover, female calls might also influence song copying^49^. Understanding this influence better might provide additional context for understanding how social interactions influence rhythm copying.

Overall, our findings highlight the complex interplay between rhythm, learning, and innovation in animal communication, with implications for understanding song evolution and potential links to female preferences.

## Supplementary Information

Below is the link to the electronic supplementary material.

Supplementary Material 1

## Data Availability

Data is provided at 10.5281/zenodo.14779410.
