# Supplementary material for: Zebra finch tutees not only share the melody but also the rhythm of their tutor’s song
Source: Sci Rep. 2025 Oct 13;15:35573. doi: 10.1038/s41598-025-22811-8 (PMC12518813; doi:10.1038/s41598-025-22811-8)
Supplement: Supplementary file 1 — Supplementary Material 1 [file 41598_2025_22811_MOESM1_ESM.pdf]

Supplements for

Zebra Finch Tutees not only Share the Melody but also the Rhythm of Their Tutor's Song

Lara S. Burchardt[+*1,2,3], Judith M. Varkevisser[+*4,5], Michelle J. Spierings[*4,5,6]

[+] These authors share first authorship

1 Humboldt-Universität zu Berlin, Berlin, Germany
2 Leibniz Zentrum für Allgemeine Sprachwissenschaften Berlin, Berlin, Germany
3 Radboud University Nijmegen, Nijmegen, Netherlands
4 Institute of Biology Leiden, Leiden University, Leiden, The Netherlands
5 Leiden Institute for Brain and Cognition, Leiden University, Leiden, The Netherlands
6 Department of Behavioral and Cognitive Biology, Vienna University, Vienna, Austria

***Corresponding authors**

Email: lara.sophie.burchardt@hu-berlin.de | j.m.varkevisser@biology.leidenuniv.nl

| m.j.spierings@biology.leidenuniv.nl

ORCID: LSB: 0000-0002-9210-7934 | JMV: 0000-0001-7706-1520 | MJS: 0009-0004-7458-7251

This PDF file includes:

Supplemental Text

S Table 1 to 5

S Figure 1

S Table 1: Number of Tutees per Nest

Nest	Number of Tutees per Nest
N01	4
N02	3
N03	1
N04	4
N05	1
N06	3
N07	1
N08	3
N09	2
N10	1
N11	2
N12	2
N13	2
N14	2
N15	4
N16	1
N17	1

Detailed Statistics

We tested the differences between the different groups via Welsh's t-test, applying Bonferroni correction for multiple testing. The following number of sequences were considered per group n tutors: 168, n tutees everything: 369, n tutees all-shared: 68, tutees part-shared: 230, n tutees not-shared: 145.

S Table 2: Testing the IOI beat - Summary of t-tests and Cohen's d

Group 1	Group 2	p	**adjusted p (Bonferroni)**	**Cohen's D**
Tutor	Everything	0.28	**1.00**	**0.1**
Tutor	Part-shared	0.0000003	**0.0000038**	**-0.46**
Tutor	All-shared	0.0000016	**0.0000181**	**0.72**

Tutor	Not-shared	0.96	**1.00**	**0.01**
Everything	Part-shared	0.00	**0.0000003**	**-0.59**
Everything	All-shared	0.000013	**0.00014**	**0.55**
Everything	Not-shared	0.51	**1.00**	**-0.08**
Part-shared	All-shared	0.00	**0.00**	**0.61**
Part-shared	Not-shared	0.0000017	**0.000019**	**0.44**
All-shared	Not-shared	0.00010	**0.0011**	**-0.49**

S Table 3: Testing the Coefficient of Variation - Summary of t-tests and Cohen's d

Group 1	Group 2	p	**adjusted p (Bonferroni)**	**Cohen's D**
Tutor	Everything	0.45	**1.00**	**-0.07**
Tutor	Part-shared	0.00	**0.00**	**-0.56**
Tutor	All-shared	0.49	**1.00**	**0.12**
Tutor	Not-shared	0.0000004	**0.0000048**	**-0.61**
Everything	Part-shared	0.00	**0.0000001**	**-0.58**
Everything	All-shared	0.30	**1.00**	**0.18**
Everything	Not-shared	0.0000016	**0.000017**	**-0.55**
Part-shared	All-shared	0.0000039	**0.000043**	**0.55**
Part-shared	Not-shared	0.14	**1.00**	**0.14**
All-shared	Not-shared	0.00018	**0.0020**	**-0.58**

S Table 4: Testing the npvi - Summary of t-tests and Cohen's d

Group 1	Group 2	p	**adjusted p (Bonferroni)**	**Cohen's D**
Tutor	Everything	0.000029	**0.00032**	**-0.35**
Tutor	Part-shared	0.00	**0.00**	**-0.88**
Tutor	All-shared	0.75	**1.00**	**-0.05**
Tutor	Not-shared	0.00	**0.00**	**-1.22**
Everything	Part-shared	0.00	**0.00**	**-0.75**
Everything	All-shared	0.027	**0.30**	**0.28**
Everything	Not-shared	0.00	**0.00**	**-1.12**
Part-shared	All-shared	0.00	**0.00**	**0.75**
Part-shared	Not-shared	0.046	**0.50**	**-0.21**
All-shared	Not-shared	0.00	**0.00**	**-1.01**

S Table 5: Correlations between Rhythm Parameters and Number of Elements in the analysis unit.

	npvi	unbiased CV	IOI Beat	n Elements
npvi		**0.73**	-0.05	**-0.46**
unbiased CV	**0.73**		-0.19	**-0.25**
IOI Beat	-0.05	-0.19		-0.16
n Elements	**-0.46**	-0.25	-0.16	

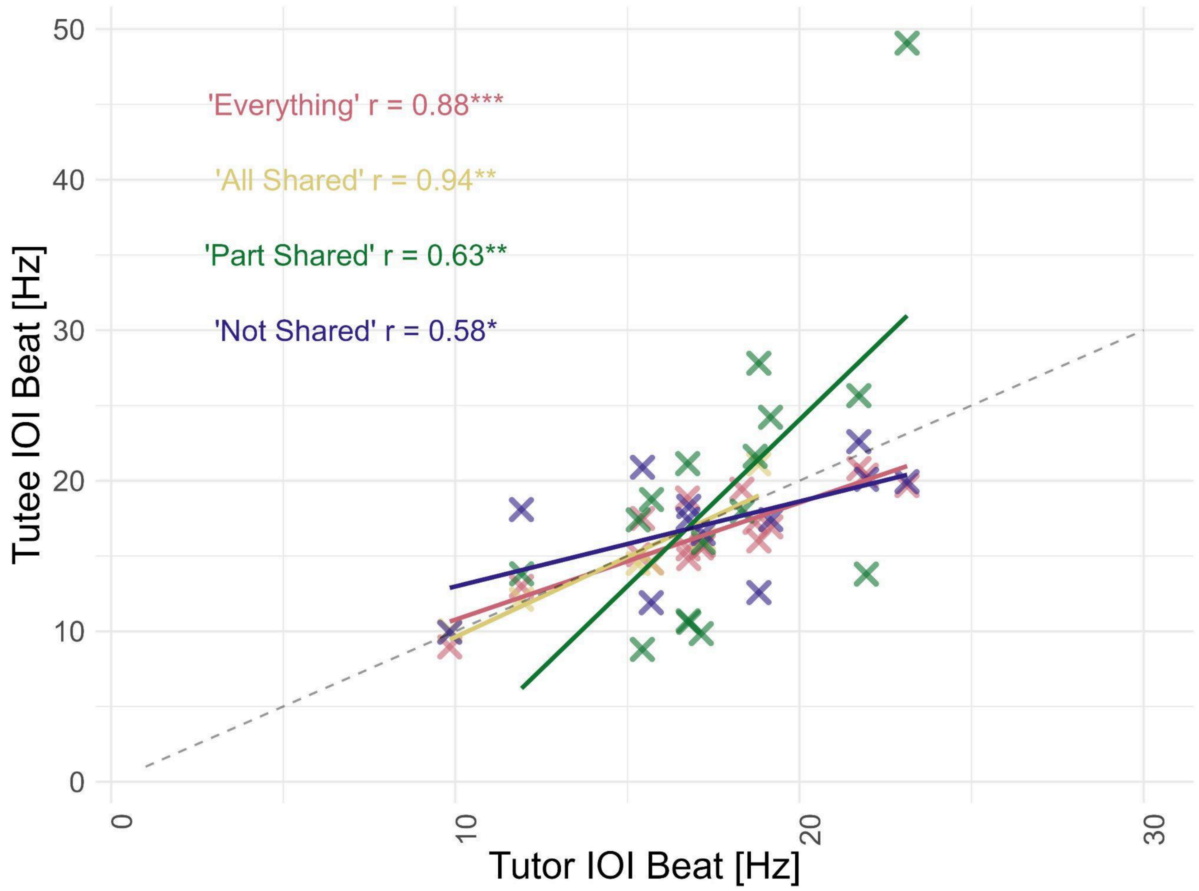

S Figure 1: The mean of each tutor's IOI beat is plotted against their tutees' mean IOI beat per nest and data subset (large X, different colors indicate different datasubsets) Dark red: "everything", same as in Figure 3B; Yellow: "All shared"; Dark Green: "Part Shared"; Purple: "Not Shared". Tutee rhythms follow their tutor's rhythms with different precision, depending on the data subset. The dotted grey line indicates a theoretical perfect match of tutor and tutee rhythms. The colored lines indicate the actual correlation of tutor and tutee rhythm. Pearson's correlation coefficient r is given in the figure for each data subset.
